# Desalination Processes’ Efficiency and Future Roadmap

**DOI:** 10.3390/e21010084

**Published:** 2019-01-18

**Authors:** Muhammad Wakil Shahzad, Muhammad Burhan, Doskhan Ybyraiymkul, Kim Choon Ng

**Affiliations:** Water Desalination and Reuse Centre, King Abdullah University of Science & Technology, Thuwal 23955-6900, Saudi Arabia

**Keywords:** standard primary energy, primary energy, standard universal performance ratio, desalination

## Abstract

For future sustainable seawater desalination, the importance of achieving better energy efficiency of the existing 19,500 commercial-scale desalination plants cannot be over emphasized. The major concern of the desalination industry is the inadequate approach to energy efficiency evaluation of diverse seawater desalination processes by omitting the grade of energy supplied. These conventional approaches would suffice if the efficacy comparison were to be conducted for the same energy input processes. The misconception of considering all derived energies as equivalent in the desalination industry has severe economic and environmental consequences. In the realms of the energy and desalination system planners, serious judgmental errors in the process selection of green installations are made unconsciously as the efficacy data are either flawed or inaccurate. Inferior efficacy technologies’ implementation decisions were observed in many water-stressed countries that can burden a country’s economy immediately with higher unit energy cost as well as cause more undesirable environmental effects on the surroundings. In this article, a standard primary energy-based thermodynamic framework is presented that addresses energy efficacy fairly and accurately. It shows clearly that a thermally driven process consumes 2.5–3% of standard primary energy (SPE) when combined with power plants. A standard universal performance ratio-based evaluation method has been proposed that showed all desalination processes performance varies from 10–14% of the thermodynamic limit. To achieve 2030 sustainability goals, innovative processes are required to meet 25–30% of the thermodynamic limit.

## 1. Introduction

The world’s demand for increasingly scarce water is escalating rapidly, challenging its accessibility for the life cycle and putting the global population at risk. The increase in water demand is mainly due to the rapid growth of population and economic development. Water security underpins the life cycle, economic growth and sustainability all over the world. In 2000, overall world water demand was 4000 billion cubic meter and it is estimated to increase over 58% by 2030. The water demand is expected to be much higher in developing countries, where over 93% additional water projections are estimated as compared to developed countries [1,2,3,4,5,6].

**Figure 1:**
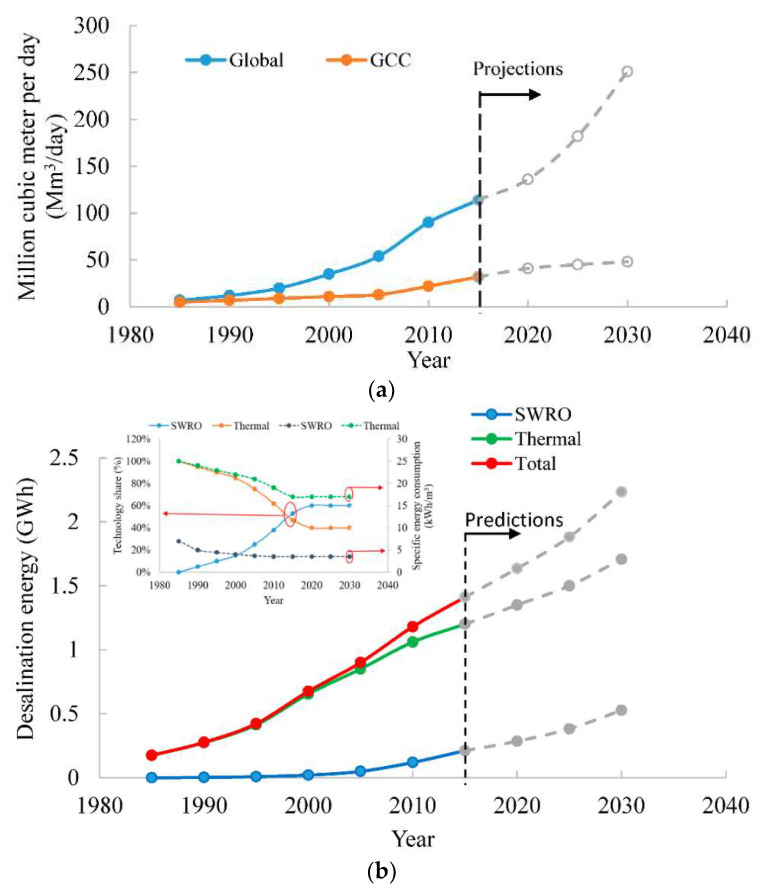
(**a**) World and Gulf Cooperation Countries (GCC) desalination capacities trend from 1985 to 2030 and (**b**) energy consumption by desalination processes [1,2,3,4,5,6,7,8,9,10,11,12,13,14].

Conventional water sources also called renewable resource such as surface water and ground water are not able to patch up the gap between supply and demand of fresh water. This growing gap can only be filled by non-conventional and non-renewable sources such as wastewater treatment and seawater desalination. In some parts of the world, even after the application of wastewater reuse, still there is shortfall of water that only can be filled by seawater desalination processes. During last 20 years, steady growth in desalination capacities installation has been observed that is expected to rise in the near future to fulfil world water demand. Installed desalination capacities are projected to be doubled by 2030 in the Gulf Cooperation Countries (GCC) as well as in the world as shown in the Figure 1a. Presently, 150 countries are operating over 19,500 desalination plants to produce 100 million cubic meters per day to fulfill the demand of a population of 300 million throughout the world. Based on current processes, the share in the world desalination market and their respective published specific energy consumptions, seawater reverse osmosis (SWRO) 60% at 3.5 Kilowatt hours per cubic meter (kWhm-3) and thermally driven processes 40% at 17 kWhm-3, the total desalination energy consumption is predicted and presented in Figure 1b. It can be noticed that with the projected expansion of desalination capacities, the energy consumption for desalination is expected to reach to 2.4 Gigawatt hours (GWh) in 2030 as compared to only 1.4 GWh in 2018 [7,8,9,10,11,12,13,14].

**Figure 2:**
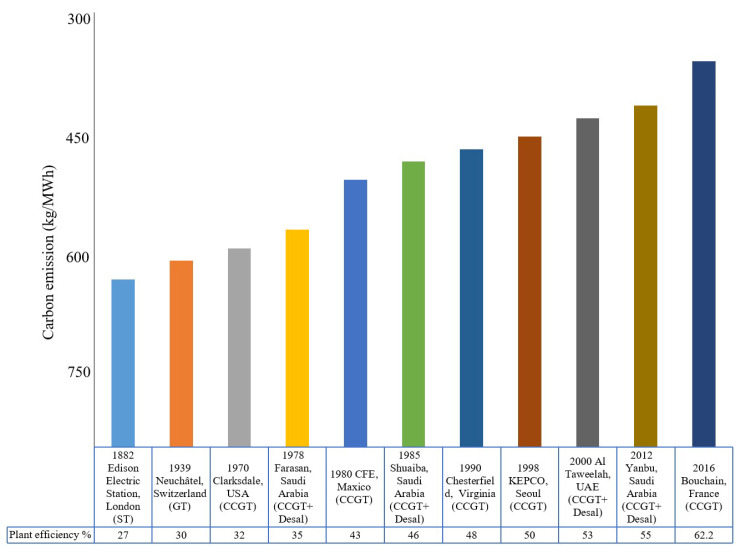
Combined cycle efficiency and environment impact trend from 1870–2018 [15,16,17,18,19,20,21,22].

The energy utilized in desalination processes is in the majority (80%) produced by thermoelectric processes that consume huge amount of water for heat rejection to complete the thermodynamic cycle. It is estimated that around 15% of water is evaporated in cooling towers to produce the required power. World electricity demand is expected to increase twofold to 34 terawatt hours (TWh) in 2035 as compared to 2010 production level. Correspondingly, inter-linked water demand is expected to increase to 790 billion cubic meters (bcm) by 2035 as compared to 583 bcm in 2010. This high water demand in thermoelectric power generation is due to low efficiencies of conventional cycles. In the past, typical power plants were operated at 28–30% energetic efficiency that is recently increased to 50% due to rapid development in combustion processes and cascading operations as shown in Figure 2 [15,16,17,18,19,20]. Recently, General Electric (GE) in partnership with EDF made history and set a Guinness World Records title for operating the world’s most efficient combined-cycle power plant at 62.22%. These gradual improvements in power plant efficiencies will help to save fossil fuel, CO_2_ emissions and additional water consumption [21,22].

It can be noticed that over a century (1880–1970), there was not much improvement in power plant efficiency due to inefficient combustion and singly operated processes. The major improvement was observed during 1970–2000 due to implementation of efficient combined cycle gas turbines (CCGT) coupled with heat recovery boiler operated steam turbines. Noticeable improvement was also observed when thermally driven desalination processes were integrated with CCGT power plants due to better utilization of low pressure steam in water production before dumping into the condenser. Today, combined CCGT and desalination processes are considered as most efficient cycle for power and water production. In conventional combined CCGT power and desalination plants, primary fuel is supplied to the gas turbine cycle where it combusts in a combustion chamber in the presence of compressed air from the compressor. The hot and high pressure gases are then expanded through the gas turbine to produce electricity. The gas turbine cycle consumes the major portion of fuel exergy due to high irreversibilities in the combustion chamber. The remaining exergy in hot exhaust gas is then recovered in the heat recovery steam generator (HRSG) to produce high pressure and temperature steam for the steam turbine cycle. Typically, three turbines, namely high pressure, medium pressure and low pressure, are arranged in cascading manners to maximize the steam potential utilization [23]. In the combined arrangement, low-pressure steam is bled from last stage of the low-pressure turbine to operate the seawater desalination cycle. These integrations improve the overall cycle performance because the steam that bled from the last stage of low pressure turbine already utilized its maximum potential but can still be useful for low-pressure desalination processes. This excellent thermodynamic synergy of process integration can only be found in combined CCGT power and thermally driven desalination processes [24]. Although the combined power and water production scheme is favorable, their analysis is more tedious due to different grades of energy utilization by the processes. There are lot of publications on combined CCGT power and desalination cycle analysis based on energetic and exergetic analysis but none is widely accepted due to limitations with all existing methodologies. The energetic approach is unable to capture the quality of working fluid utilized by a process and hence provides unfair apportionment of primary fuel in combined cycle arrangements. On the other hand, the exergy analysis can capture quantity as well as quality of working fluid utilized in any process and hence provide an accurate fuel distribution across all processes.2. Published Approach’s Limitations

There is plethora of literature available on CCGT power and desalination plants’ thermodynamic analysis presenting various methodologies and different results. Most of the literature indicates that gas turbines (GT) cycle consumes 75 ± 1% of input fuel exergy and remaining 25 ± 1% is recovered in heat-recovery steam generator (HRSG) via exhaust gases to produce steam for bottoming steam turbine cycle. The steam turbines consume another 20% of remaining fuel exergy and bled steam carry only 4 ± 1% exergy for desalination cycles. The remaining exergy is dumped into the condenser in the form of dead steam and lost in process irreversibilities [25,26,27,28].

In terms of desalination processes analysis, most of the literature presented quantitative or energetic approaches since the 1970s. For example, Wade [29] presented five different schemes of power and desalters including thermal and membranes and the cost was analyzed based on energy consumed and power plant efficiency. Kamal [30] emphasized an energetic approach to compute the payback for combine power and water production cycle. Saeed [31] estimated the proportions of fuel utilized by each processes in a combined cycle based on a simple efficiency approach, output to input ratio. Osman et al. [32] proposed energetic approach for cost estimation of a combine power and water system. They conducted series of experiments to optimize the operational parameters of a boiler for the highest efficiency. Al-Sofi et al. [33] presented first law of thermodynamics for dual-purpose plant fuel cost allocation. They simply applied output to steam flow rate ratio for fuel cost distribution. Mussati et al. [34] suggested a cost subtraction method in which the one product cost is predetermined based on annual credit method and second output cost is calculated by subtraction. Darwish [35] recommended fuel saving methodologies by four different schemes of individual plant operation. He also highlighted the boiler optimization to adjust as per plant requirements. Wang et al. [36] proposed an energetic approach based on input and outputs and also some exergetic analysis based on output. Helal [37] and Lozano et al. [38] demonstrated energetic approach for tri-generation system (power, cooling and desalination) cost apportionment. All these conventional quantitative approaches can be only applied for comparison of the same energy input processes such as multi-effect desalination (MED) to MED, multi-stage flash (MSF) to MSF, and SWRO to SWRO. The cross processes comparison needed quantitative as well as qualitative analysis. This can be achieved by invoking the 2nd Law of thermodynamics and exergetic analysis approaches.

Although some authors have highlighted the exergy accounting concept, all calculations were performed using the energetic input of steam to the total output produced. Plenty of definitions are proposed by the researchers for a desalination processes efficiency calculation based on second law of thermodynamics [39,40,41,42,43,44,45]. The literature mostly showed that the exergetic approaches were only applied to individual system performance analysis, not for combined cycle overall cost apportionment. For example, Hosseini et al. [46] explained a total revenue approach for economic analysis of dual-purpose plants. They utilized exergy to investigate the system components’ efficiency. Mistry et al. [47,48,49] presented second law analysis of desalination processes and ignored the current best practices, combined power and water production for the best thermodynamic synergy. Such approaches may be correct for stand-alone desalination processes but it did not reflect the chorological evolvement of combine cycles to achieve current best efficiency. Similarly, Lienhard et al. [50] outlined an exergetic analysis of desalination processes by considering them as a black box. They derived a second law efficiency and thermodynamic limit by using Carnot work approach. Again, the analysis was presented as a stand-alone system without combining it with the power-generation system to represent real production practices. Fitzsimons et al. [51] conducted detailed review of over sixty published articles on exergetic analysis. They showed that all articles are based on stand-alone desalination processes analysis with over 80% variations in the results due to different approached and assumptions.

It can be noticed that, there are two major gaps in published literature. Firstly, most of the literature is based on the conventional energetic approach for combined power and desalination processes analysis that provides unfair apportionment of primary fuel by ignoring the grade of energies utilized by the processes. Secondly, there is no common platform to compare all desalination processes by incorporating different grades of energies in the CCGT arrangement. Even within thermally driven processes such as MED and MSF the activation steam temperatures are different. Conventionally, desalination processes are presented based on different kinds of energy for comparison purposes such as electricity (kWh) and thermal (kWh). Even though the units are same, this comparison is not fair as grade of energies are different. In this paper, we develop a detailed thermodynamic frame work based on a standard primary energy (SPE) approach to resolve two main issues, namely; (i) an accurate apportionment of primary fuel exergy across each processes in a combined cycle arrangement based on their operational parameters; and (ii) comparison of all desalination processes at a common platform called the standard universal performance ratio (SUPR), by converting different types and grades of energies to standard primary energy. This can be achieved by invoking the second law of thermodynamics where the primary energy can be supplied to achieve the same equivalent work of the separation processes. The proposed approach circumvents the deficiency of derived energy units (kWh) used singly, as these energy units omit the quality of supplied energy.

The SPE approach consider meaningful temperature ratios to complete thermodynamic cycle, from the adiabatic flame temperature to the ambient reservoir. The proposed SPE methodology has two requisites. Firstly, it is important to consider the current best available practice of power and water production. Secondly, the operating inlet and out temperatures of actual separation processes in the form of work or heat should be normalized to a common standard inlet and outlet temperatures. The detailed methodology is presented in the following sections.

## 2. Thermodynamic Framework for Standard Primary Energy (SPE)

**Figure 3:**
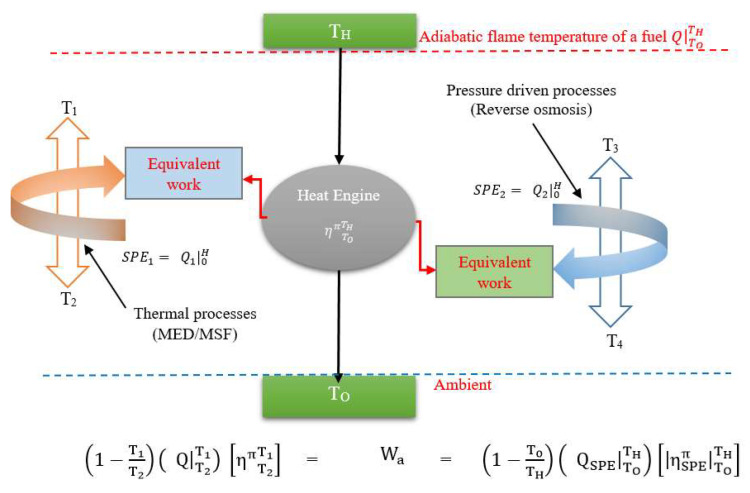
The standard primary energy (SPE) concept to emulate actual desalination processes.

The separation processes can be modelled as a heat engine operating between heat source temperature (T_1_) and reservoir temperature (T_2_). The heat engine will extract the same amount of derived energy $Q{|}_{T_{2}}^{T_{1}}$ as the separation process and produce an arbitrary work (W_a_) as shown in Figure 3.

The derived energies utilized by the separation processes can be different in grades, so the equivalent work approach is applied, where the simulated heat engine will produce the same arbitrary work by operating between defined temperature limits of the standard primary energy (SPE). This is performed by invoking the second law efficiency where the standard primary energy ($Q_{\mathrm{SPE}}{|}_{T_{o}}^{T_{H}}$) for the same work output can now be determined at the common platform conditions. The second law efficiency is defined as the ratio of actual work (W_a_) to the ideal work of a separator supplied with the derived energy $Q{|}_{T_{2}}^{T_{1}}$. The heat driven desalination process can be normalized to a standard primary energy (SPE), ${\left(Q{|}_{T_{o}}^{T_{H}}\right)}_{\mathrm{MED}}$, working across a common temperature levels where the heat it is assumed to produce the equivalent work of the MED. Similarly, the work-driven reverse osmosis (RO) process can also be normalized to the common energy units, i.e., the SPE, ${\left(Q{|}_{T_{o}}^{T_{H}}\right)}_{\mathrm{RO}}$ in the same framework. Hence, by transforming thermodynamically into the SPE all forms of derived energy supplied to the desalination processes, such as the work-driven or the heat driven separation methods, a common platform for efficacy comparison is established for all desalination methods that are supplied with assorted types of secondary or derived energies.

### Conversion Factors for Derived Energies

**Figure 4:**
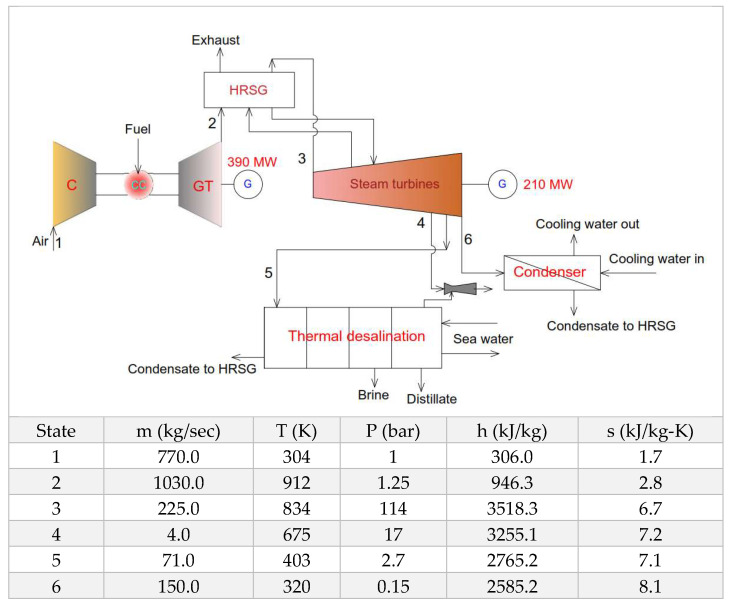
Typical combined power and desalination system schematic and state points.

To develop conversion factors from derived energy to primary energy based on the equivalent work approach, the proposed thermodynamic framework is applied to a combined power and desalination scheme. The typical CCGT + Desalination flow schematic with state points is presented in Figure 4 and detailed thermodynamic framework is summarized in Table 1. Using the thermodynamic state points of the flow diagrams and thermodynamic framework, exergetic proportions are calculated for each component of the cycle.

It was found that the Brayton cycle consumes 58.32% exergy of input fuel and exhaust gases at 639°C are dumped into the heat recovery steam generator (HRSG) for additional exergy re-utilization. Steam from the HRSG is directed to Rankine cycle to produce additional power and all three turbines consume 38.93% of input fuel exergy. It also includes internal losses and proportion of steam exergy that condensed into the condenser. The bleed steam to fulfill the requirement of MED water production only carries 2.75% of fuel exergy that also include all related losses. These exergy proportions are translated into conversion factors (CF) for the convenience of plant operators and decision makers. These conversion factors will help to convert different kind of derived energies to standard primary energy to provided common platform for comparison of all kinds of separation processes. For example, for typical example plant, one unit of electricity needs 2.0 units of standard primary energy due to turbine and generator efficiencies. On the other hand, one unit of SPE can produce 36.36 units of low grade steam needed to operate the MED cycle. The detail of SPE and conversion factors is presented in Table 2. The major advantage of proposed SPE approach is the maximization of exergy potential by pushing the temperature limits to adiabatic flame to ambient conditions. This shows superiority over previously published primary energy approaches and other methodologies [52,53].

## 3. Standard Universal Performance Ratio (SUPR)

The conventional unfair performance parameter of desalination processes now can be transformed to a more accurate parameter based on the common platform of SPE. The new performance parameter is called the standard universal performance ratio (SUPR) as shown in Equation (1).

(1) $$\begin{array}{r} \mathrm{Standard}\;\mathrm{Universal}\;\mathrm{Performance}\;\mathrm{Ratio}\;\left(\mathrm{SUPR}\right)=\frac{\begin{array}{c} \mathrm{Equivalent}\;\mathrm{heat}\;\mathrm{of}\;\mathrm{evaporation}\;\\ \mathrm{of}\;\mathrm{distillate}\;\mathrm{production}\; \end{array}}{\mathrm{SPE}\;\mathrm{input}\;}\\ ≅\frac{2326\;\left\{\frac{\mathrm{kJ}}{\mathrm{kg}}\right\}}{3.6\;\times {\left[\mathrm{CF}1\left\{\frac{{\mathrm{kWh}}_{\mathrm{elec}}}{m^{3}}\right\}+\mathrm{CF}2\left\{\frac{{\mathrm{kWh}}_{\mathrm{ther}}}{m^{3}}\right\}+\mathrm{CF}3\left\{\frac{{\mathrm{kWh}}_{\mathrm{Renewable}}}{m^{3}}\right\}\right]}_{}} \end{array}$$

Table 3 presents the SPE and SUPR calculations based on the proposed methodology. The converted standard primary energy-based results highlighted the inadequacy of conventional reporting procedures that ignored the quality of energy supplied to cogeneration processes. It can be seen clearly that in MED processes SPE consumption is the lowest, 5.5kWh/m^3^_SPE_ followed by RO and MSF processes. Even though MED processes efficiency is the highest but still they can achieve only 14% of thermodynamic limit. It shows that all conventional desalination process are not sustainable as they only operate between 10–14% of thermodynamic limit. To achieve future sustainability goals, separation processes need to attain 25–30% of thermodynamic limit, SUPR in the range of 200–250.

**Figure 5:**
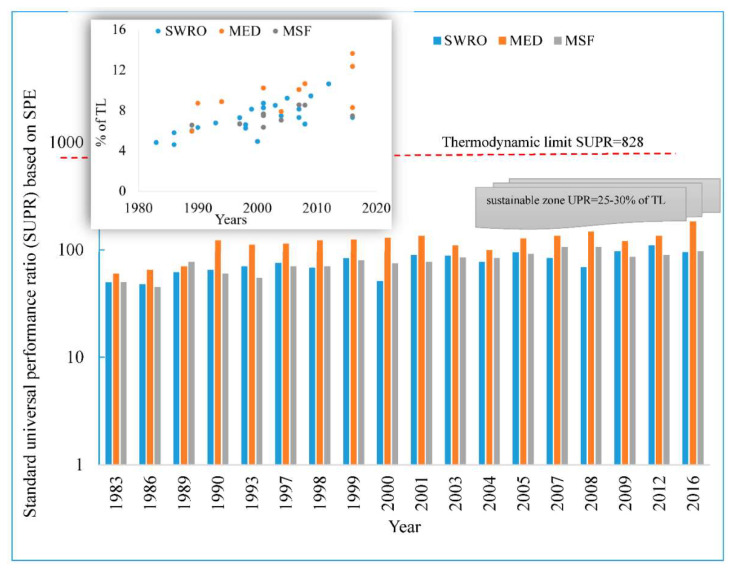
Commercial-scale seawater desalination processes performance trend from 1983–2016.

To investigate the performance trend in the past and present, over 40 desalination plant data was collected and performance parameters were calculated based on proposed SPE methodology as presented in Figure 5. It can be noticed that for the last three decades, 1983–2016, marginal improvement was observed in the performance of desalination processes. The insert shows that the performance was improved from 5% of TL to 14% of TL. This improvement is attributed to better material and pre-treatment facilities development of separation processes. These marginal improvements are not sustainable for future water supplies.

Recent hybridization trend of different desalination processes to overcome the individual processes limitations have ability to achieve SUPR ≈ 170 and energy efficiency up to 20% of TL [56,57,58,59,60,61,62,63,64,65,66,67], Kudus to the thermodynamic synergy between cycles. There is a need for more innovative processes that could maximize the input fuel exergy to boost water production, and it is plausible for process design to achieve up to 30% of TL, meeting the goals of sustainable seawater desalination.

## 4. Roadmap for Sustainable Water Supplies

**Figure 6:**
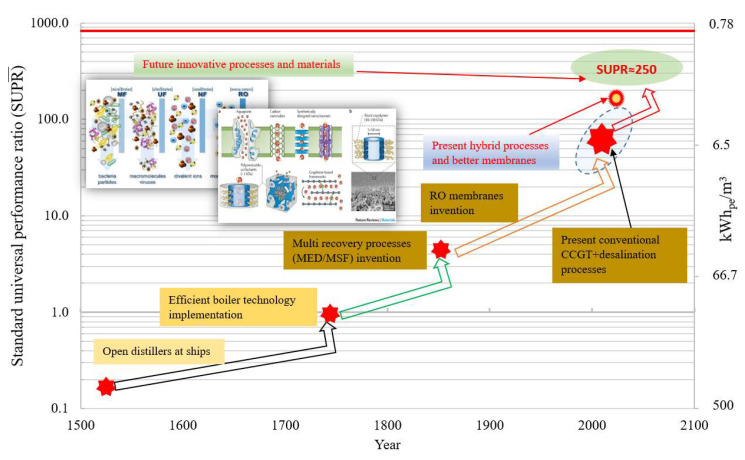
Desalination processes development since last 3 decades. The paradigm shift in technology can help to gain a quantum jump in performance.

As the all conventional desalination processes are operating far from the thermodynamic limit, innovative processes are required to achieve future sustainability goals. The proposed SPE and SUPR methodologies will help to investigate the best efficient desalination processes for future sustainability. From past experience, it has been noted that the major improvements in desalination performance can only be achieved by a paradigm shift in the technology or by thermodynamic integration of processes. The peripheral improvements of processes such as control of fouling and scaling on the key surfaces can achieve merely a gradual increment of efficiency, as can be seen in Figure 6. After almost three decades, now desalination processes are on the verge of new discoveries or “out of box” solutions to achieve a quantum jump in performance. We believe that improvement in the recent hybridization trend and highly efficient graphene-based membranes development will revolutionize the desalination industry to achieve an efficiency goal of 25–30% of TL. In addition, biodesalination that produce fresh water using biological processes and the integration of nanomaterials in the desalination process for material and system enhancement has been acknowledged to overcome the barriers and limitations that are currently facing the desalination technology can be alternative options for future sustainability [68,69].

## 5. Conclusions

Conventional derived energy-based desalination processes’ efficiency comparison methods cannot portray the essence of real efficiency. The present methodology falls short in giving a fair comparison when assorted separation processes, consuming electricity and low-grade steam, are involved. A common platform, based on the second law of thermodynamics and hence the second law efficiency, has been demonstrated in this article that equitably translates the derived-energy to the common platform of the SPE. It shows that the desalination processes consume only 2.75% of primary fuel exergy in combined cycle arrangements. A new figure of merit, called the SUPR, is developed that captures the energy efficacy accurately. Currently, all desalination processes are operating only at SUPR values of 92–118, corresponding to 10–14% of TL. To approach higher efficiency levels, the utilization of input fuel exergy must be maximized with excellent thermodynamic synergy between processes to achieve 25–30% of TL. For future sustainability, alternative, new “out-of-box” solution(s) are needed. In closing, the shortcomings of quantifying the derived energy alone have been clarified. A thermodynamically rigorous method, namely translation from the derived or secondary energy to a common platform of standard primary energy (SPE) consumption, is absolutely necessary in providing an effective comparison of all desalination processes.

## Figures and Tables

**Table 1:** Combined cycle flow schematic and operational parameters. Proposed methodology to calculate conversion factors and performance parameters.

**Brayton Cycle (GT)**	
**Carnot work**	$W_{\mathrm{carnot}}^{\mathrm{GT}}=\left(1-\frac{T_{o}}{T_{H}^{0}}\right)Q_{H}^{o}$
Second law efficiency	$\eta_{\Xi ,\;\mathrm{GT}}^{\pi }=\frac{\Xi_{a}}{\Xi_{\mathrm{rev}}}\approx \;\frac{{\;W}_{a}}{W_{\mathrm{rev}}^{\mathrm{GT}}}$
Exergy utilization factor for GT cycle	$\%\;\mathrm{of}\;\mathrm{Exergy}\;\mathrm{utilization}\;=\;\frac{W_{\mathrm{carnot}}^{\mathrm{GT}}}{Q_{H}^{o}}+\%\;\mathrm{of}\;\mathrm{UL}$
Standard primary energy conversion	$W_{\mathrm{carnot}}^{\mathrm{GT}}=\left(Q_{\mathrm{SPE}}^{\mathrm{GT}}\right)\left(1-\frac{T_{o}}{T_{\mathrm{adia}}}\right)\eta_{\Xi ,\;\mathrm{SPE}}^{\pi }$
**Rankine Cycle (STs)**	
Carnot work	$W_{\mathrm{carnot}}^{\mathrm{ST}}=\left(1-\frac{T_{o}}{T_{H}^{0}}\right)\left(Q_{H}^{o}-Q^{\mathrm{GT}}\right)$
Second law efficiency	$\eta_{\Xi ,\;\mathrm{ST}}^{\pi }=\frac{\Xi_{a}}{\Xi_{\mathrm{rev}}}\approx \;\frac{{\;W}_{a}}{W_{\mathrm{rev}}^{\mathrm{ST}}}$
Exergy utilization factor for ST cycle	$\%\;\mathrm{of}\;\mathrm{Exergy}\;\mathrm{utilization}\;=\;\frac{W_{\mathrm{carnot}}^{\mathrm{ST}}}{\left(Q_{H}^{o}-Q^{\mathrm{GT}}\right)}\;+\%\;\mathrm{of}\;\mathrm{UL}$
Standard primary energy conversion	$W_{\mathrm{carnot}}^{\mathrm{ST}}=\left(Q_{\mathrm{SPE}}^{\mathrm{ST}}\right)\left(1-\frac{T_{o}}{T_{\mathrm{adia}}}\right)\eta_{\Xi ,\;\mathrm{SPE}}^{\pi }$
**Desalination Cycle**	
Carnot work for separation	For the same equivalent work, the standard primary energy is given by: $W_{\mathrm{carnot}}^{\mathrm{Sep}}=\left(Q_{\mathrm{SPE},}^{\mathrm{sep}}\right)\left(1-\frac{T_{o}}{T_{\mathrm{adia}}}\right)\eta_{\Xi ,\;\mathrm{SPE}}^{\pi }$
Second law efficiency of separation	$\eta_{\Xi ,\;\mathrm{sep}}^{\pi }=\frac{W_{\mathrm{carnot}}^{\mathrm{Sep}}}{\left(Q_{a,}^{\mathrm{sep},{\;T}_{\mathrm{sep}}}\right)\left(1-\frac{T_{o}}{T_{\mathrm{sep}}}\right)}$
Actual separation work	$W_{\mathrm{actual}}^{\mathrm{Sep}}=\frac{W_{\mathrm{carnot}}^{\mathrm{Sep}}}{\eta_{\Xi ,\;\mathrm{SPE}}^{\pi }}$
SPE proportions for separation processes	$SPE=\frac{Q_{\mathrm{SPE}}^{\mathrm{sep},}}{Q_{H}^{o}}+\%\;\mathrm{of}\;\mathrm{UL}$
UL: unaccounted losses that includes; (a) exergy of exhaust gas leaving from HRSG, (b) GT losses, (c) STs losses and (d) exergy of steam condensed in the condenser	

**Table 2:** Summary of GT, ST and desalination plants analysis and conversion factors calculation.

	Carnot Work (MW)	Exergy Destruction (%)	Cumulative Exergy Destruction (%)
Gas turbine cycle			
**Gas turbine**	563.0	58.32	58.32
**Exhaust gas to ambient**	18.5		
**Un-accounted losses share**	21.0		
**Sub-total**	602.5		
**2nd law efficiency**	64.5%		
Steam turbine cycle			
**Steam turbines**	298.5	38.93	97.25
**Re-heating**	22.0		
**HRSG losses share**	51.5		
**Condenser losses share**	19.7		
**Un-accounted losses share**	10.5		
**Sub-total**	402.2		
**2nd law efficiency**	50.1%		
Multi effect desalination cycle			
**Multi-effect desalination (MED) heat source**	18.0	2.75	100
**Thermal vapor compressor (TVC)**	5.29		
**HRSG losses share**	3.44		
**Condenser losses share**	1.39		
**Un-accounted losses share**	0.18		
**Sub-total**	28.3		
Conversion factors (CF) from derived energy to SPE			
**For combined cycle gas turbine (CCGT) electricity (weighted factor)**	2.0 (equivalent to 50% CCGT efficiency)		
**For MED**	36.36		

**Table 3:** SPE and universal performance ratio (UPR) calculation of major desalination processes.

Specific Energy Consumption and Performance Ratio	Reverse Osmosis (SWRO)	Multi-Stage Flashing (MSF)	Multi-Effect Distillation (MED)
Electricity (kWh__elec_ m^−3^) [54,55]	3.5	2.8	1.8
Thermal (kWh__ther_/m^−3^) [54,55]	-	95.0	68.0
Equivalent Standard Primary Energy (SPE) and standard universal performance ratio (SUPR)			
Conversion factor for electricity (weighted CF_elec_)	2.0		
Conversion factor for thermal for less than 130 °C operation (CF_ther_)	-	36.36	
Standard primary energy (Q__SPE_)	7.01	7.77	5.47
Standard universal performance ratio (SUPR)	92.30	83.15	118.12
SUPR % of thermodynamic limit	11.1%	10.0%	14.2%

## Abbreviations

GCC	Gulf Cooperation Countries
MED	multi effect desalination
MSF	multi stage flash
SRRO	seawater reverse osmosis
TWh	terawatt hours
GWh	gigawatt hours
MWh	megawatt hour
KWh	kilowatt hours
BCM	billion cubic meter
CCGT	combined cycle gas turbine
GT	gas turbine
HRSG	heat recovery steam generator
ST	steam turbine
SPE	standard primary energy
SUPR	standard universal performance ratio
TVC	thermo vapor compressor
HP	high pressure
MP	medium pressure
LP	low pressure
CF	conversion factors
TL	thermodynamic limit
W	work
UL	unaccounted losses
**Subscripts**	
Th	thermal
T_H_	adiabatic flam temperature
To	ambient temperature
TL1 to TL5	process temperatures
Elec	electric
Ther	thermal
a	arbitrary
Rev	reversible
Sep	separator
